# SETD4 Confers Cancer Stem Cell Chemoresistance in Nonsmall Cell Lung Cancer Patients via the Epigenetic Regulation of Cellular Quiescence

**DOI:** 10.1155/2023/7367854

**Published:** 2023-05-27

**Authors:** Yuehong Wang, Yuman Yu, Weijun Yang, Linying Wu, Yaoshun Yang, Qianyun Lu, Jianying Zhou

**Affiliations:** ^1^Department of Respiratory Disease, The First Affiliated Hospital, College of Medicine, Zhejiang University, Hangzhou 310003, China; ^2^Department of Geriatrics, The First Affiliated Hospital, College of Medicine, Zhejiang University, Hangzhou 310003, China; ^3^MOE Laboratory of Biosystem Homeostasis and Protection, College of Life Sciences, Zhejiang University, Hangzhou 310058, China

## Abstract

Increasing evidence indicates that quiescent cancer stem cells (CSCs) are a root cause of chemoresistance. SET domain-containing protein 4 (SETD4) epigenetically regulates cell quiescence in breast cancer stem cells (BCSCs), and SETD4-positive BCSCs are chemoradioresistant. However, the role of SETD4 in chemoresistance, tumor progression, and prognosis in nonsmall cell lung cancer (NSCLC) patients is unclear. Here, SETD4-positive cells were identified as quiescent lung cancer stem cells (qLCSCs) since they expressed high levels of ALDH1 and CD133 and low levels of Ki67. SETD4 expression was significantly higher in advanced-stage NSCLC tissues than in early-stage NSCLC tissues and significantly higher in samples from the chemoresistant group than in those from the chemosensitive group. Patients with high SETD4 expression had shorter progression-free survival (PFS) times than those with low SETD4 expression. SETD4 facilitated heterochromatin formation via H4K20me3, thereby leading to cell quiescence. RNA-seq analysis showed upregulation of genes involved in cell proliferation, glucose metabolism, and PI3K-AKT signaling in activated qLCSCs (A-qLCSCs) compared with qLCSCs. In addition, SETD4 overexpression facilitated PTEN-mediated inhibition of the PI3K-mTOR pathway. In summary, SETD4 confers chemoresistance, tumor progression, and a poor prognosis by regulating CSCs in NSCLC patients.

## 1. Introduction

Lung cancer is a leading cause of cancer-related death worldwide, and nonsmall cell lung cancer (NSCLC) accounts for approximately 85% of all lung cancers [1]. The prognosis for NSCLC is still poor. For early-stage NSCLC patients, tumor recurrence or metastasis is a crucial problem despite the use of chemotherapy and/or radiotherapy after surgery. For patients with locally advanced or metastatic NSCLC, many treatment options have been identified. Beyond conventional treatment strategies, such as chemotherapy and radiotherapy, targeted therapy and immunotherapy have become important options in the treatment of advanced NSCLC [2]. However, these therapies are unable to eradicate all cancer cells. The 5-year survival rate of NSCLC patients with distant metastases is only approximately 6% [3]. The reexpansion of drug-resistant cancer cell clones facilitates tumor relapse and progression [4]. Thus, treatment strategies targeting these drug-resistant cancer cells are crucial to improving the survival of lung cancer patients.

Previous studies have identified that a small proportion of cancer stem cells (CSCs) in NSCLC can survive chemotherapy and radiotherapy, and these cells are responsible for therapeutic resistance, relapse, and tumor metastasis [5, 6]. Accumulating evidence indicates that the root cause of drug resistance is a small subpopulation of quiescent cancer stem cells (qCSCs) [7–11]. qCSCs are maintained in a nondividing state, which enables them to resist harsh environmental conditions, including chemotherapy, radiotherapy, and hypoxia [12–14]. Importantly, this state is reversible, and qCSCs can be activated and reenter the cell cycle under favorable conditions, resulting in tumor relapse and metastasis [15, 16]. Epigenetic mechanisms such as DNA methylation, histone modifications, and some key signaling pathways including the Wnt/*β*-catenin signaling pathway, hedgehog signaling pathway, and Notch signaling pathway promote CSC activation, tumorigenesis, and metastasis [17]. Alteration of DNA methylation and histone modifications could result in an expansion of CSCs [18]. Thus, qCSCs can survive during chemotherapy, and the activation of these cells is responsible for tumor regrowth after chemotherapy [19]. Similarly, the activation of qCSCs is necessary for tumor relapse or metastasis, which occurs long after initial treatment [20]. Current therapies fail to eliminate resistant qCSCs, making them attractive therapeutic targets in cancer therapy [21].

Understanding the potential mechanism of quiescent cell maintenance is crucial to eradicating qCSCs. It has been reported that metabolic regulation and epigenetic modification are involved in stem cell homeostasis and quiescence maintenance [22]. Several signaling pathways, including TGF-*β*/SMAD [11], BMP [23], and YAP/TAZ [7–24], have been implicated in stem cell quiescence. Besides, PTEN, a negative regulatory factor of the PI3K-AKT pathway, has been reported to regulate cell quiescence in stem cells [25]. PTEN was necessary for quiescence maintenance in hematopoietic stem cells (HSCs) [26–28]. PTEN inhibition and subsequent activation of PI3K-AKT-mTOR were reported to increase the proliferative activity of HSCs [29]. However, little is known about the original determinants of stem cell quiescence. *Artemia* is a suitable model system to study cell quiescence [30, 31]. Dai et al. [32] previously used *Artemia* diapause embryos as a study model and identified SET domain-containing protein 4 (SETD4) as the key regulator of cell quiescence during diapause formation in *Artemia*. SETD4 regulates cell quiescence through heterochromatin formation by catalyzing the trimethylation of lysine 20 of histone 4 (H4K20me3) [32]. On this basis, they extended this finding to CSCs and found that SETD4 regulated cell quiescence in CSCs in the same manner [33]. In addition, SETD4-defined quiescent breast cancer stem cells (qBCSCs) were resistant to chemoradiotherapy in vitro, and the expression level of SETD4 was increased after chemotherapy in a xenograft tumor model, indicating that SETD4 may be correlated with chemoresistance [33].

However, it remains unclear whether SETD4 could be an indicator of quiescent CSCs in different tumor types, and it also needs to be further verified whether SETD4-positive quiescent CSCs are correlated with tumor progression and prognosis. In this study, we assessed SETD4 expression and analyzed the relationship between SETD4 expression and chemoresistance and prognosis in NSCLC patients.

## 2. Materials and Methods

### 2.1. Cell Line Cultures

The human lung cancer cell lines NCI-H1299 and NCI-H520 were obtained from the American Type Culture Collection (ATCC; Manassas, VA, USA) and cultured in RPMI-1640 with 10% FBS at 37°C in a humidified 5% CO2 incubator.

### 2.2. Chemicals and Antibodies

Taxol (TAX) was purchased from Sangon. Cisplatin (CDDP), pemetrexed (PEM), and gemcitabine (GEM) were purchased from Selleck Chemicals. Antibodies against the following target antibodies were used: SETD4 (Sigma–Aldrich; HPA024073 for immunofluorescence (IF) assays and Santa Cruz Biotechnology; sc-514060 for western blotting (WB) assays), Ki67 (Cell Signaling Technology; 9449S for IF assays and Abcam; ab16667 for WB assays), PCNA (Abcam; ab18197), H4K20me3 (Cell Signaling Technology; 5737S), H3K9ac (Abcam; ab10812), HP1-*α* (Cell Signaling Technology; 2616S), GAPDH (Cell Signaling Technology; 2118S), H3 (Abcam; ab1791), H4 (Abcam; ab10158), CD133 (HuaAn-Biotec; EM1701-28 for IF assays), CD133 (CD133 MicroBead Kit; Miltenyi Biotec; 130-100-857), CD44 (Abcam; ab254530), ALDH1 (BD Pharmingen; 611194), caspase-3 (Cell Signaling Technology; 9662S), PI-3 kinase p85*α* (Abcam; ab191606), phospho-PI-3 kinase (Cell Signaling Technology; 4228S), mTOR (Cell Signaling Technology; 2983 T), and PTEN (Cell Signaling Technology; 9188S).

### 2.3. Clinical Sample Acquisition

This study included 166 tumor specimens from patients with histologically confirmed stages I-IV NSCLC from January 2016 to July 2020 in our hospital. Clinical sample acquisition was approved by the Ethics Committee of the First Affiliated Hospital of Zhejiang University and conducted according to the Declaration of Helsinki. Most of the specimens from early-stage NSCLC patients were obtained by surgery, while specimens from advanced-stage NSCLC patients were obtained by *bronchoscopic biopsy or* percutaneous core-needle lung biopsy. Tumor specimens were fixed with 4% formalin, paraffin embedded, and sectioned at 3 *μ*m thickness for IF analysis. In addition, fresh tumor tissues were stored in tissue storage solution at 4°C and processed immediately (within 2 hours) for subsequent primary culture and chemoradiotherapy in vitro.

### 2.4. Primary Culture of Lung Cancer Cells and Chemoradiotherapy In Vitro

Surgical tumor specimens were chopped and digested in tumor dissociation solution (Miltenyi Biotec, 130-095-929) at 37°C for 1 hour. Then, the cells were filtered through 40 *μ*m cell strainers and centrifuged at 300 × g for 5 minutes. The cells were resuspended in ultralow attachment 6-well plates and cultured in DMEM/F12 (Corning; 10–092-cv) for one week. Then, these cells were treated with 200 nmol/L TAX and 10 *μ*mol/L CDDP and irradiated (30 Gy) approximately 10 days after chemotherapy initiation. The medium was replaced twice every week, and sustained drug treatment lasted for over 2 weeks. The surviving cells were obtained via a dead cell removal kit (Miltenyi Biotec; 130-090-101) according to the manufacturer's instructions and used for subsequent experiments.

### 2.5. Activation of Quiescent Lung Cancer Stem Cells (qLCSCs)

Chemoradioresistant qLCSCs were seeded at a density of 1000 cells per well in ultralow attachment 6-well plates and maintained in tumorsphere formation medium (DMEM/F12 including 2% serum replacement (SR; Thermo Fisher Scientific; 10828028), 20 ng/mL EGF (PeproTech), 5 ng/mL heparin sodium (MedChemExpress; 9041-08-1), and 20 ng/mL bFGF (PeproTech; 96-100-18B-500)) at 37°C in a 5% CO2 incubator.

### 2.6. IF Analysis

Paraffin sections were fixed with 4% formalin and embedded in paraffin, and cell samples were fixed with 4% paraformaldehyde. The samples were permeabilized with 0.25% Triton X-100 for 10 minutes and blocked with 5% donkey serum at room temperature for 1 hour. The blocked slides were incubated with appropriate primary antibodies overnight at 4°C and then with species-specific Alexa 488- or Alexa 594-conjugated secondary antibodies for 2 hours at room temperature. Subsequently, the slides were sealed and photographed under a Leica fluorescence microscope (Leica DMi8). At least 5 random visual fields with a minimum of 2000 cells were analyzed for each sample.

### 2.7. Overexpression of the SETD4 Protein

According to the sequence of the human SETD4 gene (NM_017438) in GenBank, the pLenti-EF1a-SETD4-CMV-GFP-P2A-Puro overexpression plasmid (Vigene Biosciences) was synthesized and cotransfected with two packaging vectors, psPAX2 (12260, Addgene) and pMD2.G (12259, Addgene), into HEK293T cells, and the viral supernatant was harvested 48 hours later. Then, CD133-sorted cells were infected with the filtered virus-containing supernatant in the presence of 10 *μ*g/ml ADV-HR for 48 hours.

### 2.8. WB

Cultured cells were lysed in RIPA lysis buffer (Beyotime, P0013B). Lysate concentrations were determined by the BCA Protein Assay Kit (Thermo Fisher Scientific, Waltham, MA, USA). Equal amounts of proteins were loaded on SDS–PAGE gels and then transferred to nitrocellulose membranes. The membranes were blocked with a 5% skim milk solution at room temperature for 1 hour and incubated at 4°C overnight with primary antibodies. Images were taken and analyzed using a Bio-Rad system.

### 2.9. RNA-Seq Analysis

We isolated qLCSCs and activated qLCSCs (A-qLCSCs) from the H1299 cell line. Total RNA was extracted from qLCSCs and A-qLCSCs using the TRIzol reagent and then used for mRNA-Seq, which was performed by Novogene (Beijing, China). Differential expression analysis was implemented by the DESeq2 R package (1.16.1). Gene Ontology (GO) enrichment analysis of differentially expressed genes (DEGs) was performed using the clusterProfiler R package. Gene set enrichment analysis (GSEA) was performed using the local version of the GSEA tool (http://www.broadinstitute.org/gsea/index.jsp).

### 2.10. Statistical Analysis

For quantification, at least three independent, repeated experiments were performed. For quantification of clinical samples, at least 5 random visual fields with a minimum of 2000 cells were analyzed for each sample by ImageJ software. Statistical analysis and graphic presentation were carried out using GraphPad Prism version 7.0 (GraphPad Software, San Diego, CA, USA) and SPSS 23.0 software (IBM Corporation, Chicago, IL, USA). The data are calculated as the mean ± SD or mean ± SEM. Statistical comparisons were performed using two-tailed Student's *t* tests. Progression-free survival (PFS) was defined as the time from the beginning of chemotherapy to the date that progressive disease (PD) was observed. Survival analysis was performed using Kaplan–Meier curves and log-rank tests. A Pearson *r* correlation analysis was performed to assess the correlation between the proportion of SETD4-positive cells and PFS time. *p* < 0.05 was considered to indicate a statistically significant difference.

## 3. Results

### 3.1. SETD4-Positive Lung Cancer Cells Were Identified as qLCSCs in Tumor Specimens from NSCLC Patients

**Figure 1:**
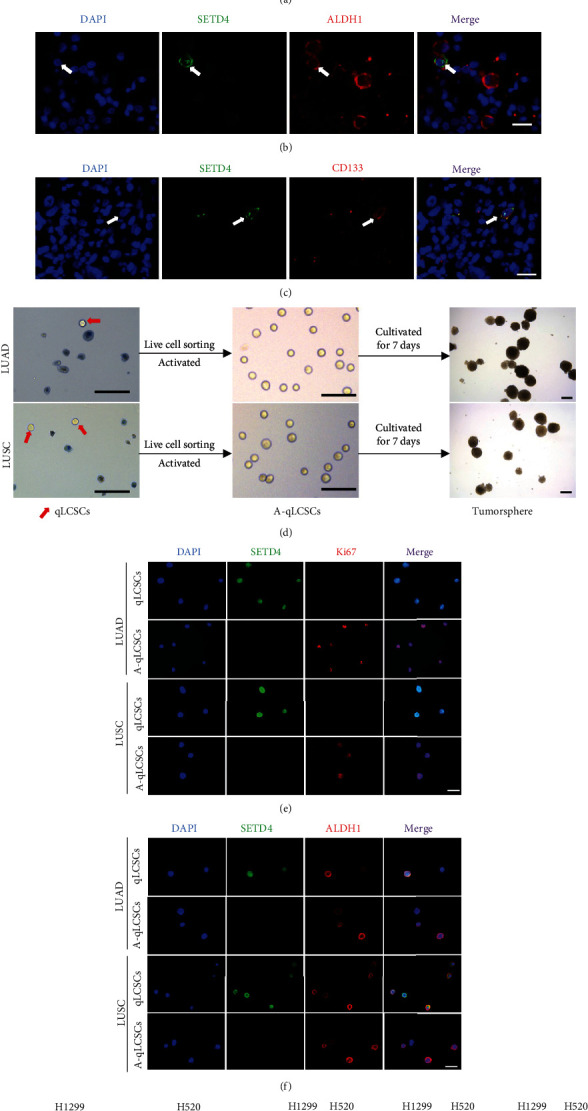
Characterization and enrichment of SETD4 in quiescent lung cancer stem cells (qLCSCs) from clinical nonsmall cell lung cancer (NSCLC) specimens. Representative immunofluorescence (IF) images of SETD4/Ki67 (a), SETD4/ALDH1 (b), and SETD4/CD133 (c) in tumor tissues from NSCLC patients. White arrow indicated SETD4-positive cells. DAPI, nuclear counterstaining. Scale bar, 20 *μ*m. (d) Isolation of qLCSCs and A-qLCSCs from clinical lung adenocarcinoma (LUAD) and lung squamous cell carcinoma (LUSC) specimens. The left panel shows representative trypan blue staining images of primary patient-derived lung cancer cells after treatment with chemotherapy and radiotherapy. Dead cells were stained with trypan blue. Red arrows indicate surviving cells, which we referred to as qLCSCs. Then, qLCSCs were activated in tumorsphere formation medium after drug withdrawal for 48 hours. The middle panel shows a representative bright field image of A-qLCSCs. The right panel shows the tumorspheres formed by A-qLCSCs after cultivation for 7 days. Scale bar, 100 *μ*m. Representative IF images of SETD4/Ki67 (e) and SETD4/ALDH1 (f) in qLCSCs and A-qLCSCs derived from clinical LUAD and LUSC specimens. DAPI, nuclear counterstaining. Scale bar, 50 *μ*m. (g) Protein levels of SETD4, Ki67 and PCNA in qLCSCs and A-qLCSCs derived from the H1299 cell line, as detected by WB analysis. *n* = 3. GAPDH was used as an internal reference. The data are presented as the mean ± SD. ^∗∗^*p* < 0.01; ^∗^*p* < 0.05.

We first characterized SETD4-positive cancer cells in NSCLC patient specimens. Ye et al. previously confirmed that SETD4 was enriched in qBCSCs from breast cancer cell lines [33]. Consistently, IF analysis in NSCLC patient specimens revealed that approximately 97% of SETD4-positive lung cancer cells lacked a Ki67 signal (Figure 1(a)), suggesting that SETD4-positive cells were maintained in a quiescent state. Additionally, most of these SETD4-positive cells showed positive ALDH1 staining (125 ALDH1^+^ cells/135 SETD4^+^ cells) (Figure 1(b)), positive CD133 staining (100 CD133^+^ cells/111 SETD4^+^ cells) (Figure 1(c)), and positive CD44 staining (80 CD44^+^ cells/91 SETD4^+^ cells) (Figure S1), which are well-established features of CSCs; thus, SETD4-positive cells were identified as CSCs. We next tried to isolate qLCSCs from clinical tumor specimens according to methods established in our previous studies [33]. We obtained 34 surgical tumor specimens derived from NSCLC patients (23 cases of lung adenocarcinoma (LUAD) and 11 cases of lung squamous cell carcinoma (LUSC)) and dissociated the cancer cells. Primary lung cancer cells were treated with chemotherapy and radiotherapy. Trypan blue staining showed a small proportion of surviving cells in LUAD and LUSC cultures after chemoradiotherapy, and we selected these cells by live cell sorting for further analysis (Figure 1(d)). Quiescent CSCs can be activated, and these cells have been proven to play a major role in tumor repopulation and relapse [34, 35]. Hence, we collected activated qLCSCs (A-qLCSCs) after inducing activation in tumorsphere formation medium after drug withdrawal for 48 hours (Figure 1(d)). Interestingly, A-qLCSCs exerted strong tumorsphere-forming properties (Figure 1(d)), suggesting their extraordinary potential for facilitating tumor relapse. We also performed tumorsphere formation assay in H1299 cell lines and A-qLCSCs derived from H1299 cell lines. Tumorspheres more than 30 *μ*m in size are calculated, and A-qLCSCs exhibited remarkable tumorsphere-forming capacity (Figure S2). Consistent with our previous study, we found that a large proportion of the surviving cells were SETD4-positive and Ki67-negative (Figure 1(e)) and exhibited high expression of cancer stem cell markers, including ALDH1 (Figure 1(f)) and CD133 (Figure S3), supporting the notion that they were quiescent LCSCs. However, we observed low expression levels of SETD4 and high expression levels of Ki67 (Figure 1(e)), ALDH1 (Figure 1(f)), and CD133 (Figure S3) in A-qLCSCs. Then, we isolated qLCSCs and A-qLCSCs from the H1299 cell line and the H520 cell line following the protocols described above. WB analysis showed that SETD4 was highly expressed in qLCSCs and downregulated in A-qLCSCs (Figure 1(g)). Concomitantly, proliferative markers, including Ki67 and PCNA, were significantly upregulated in A-qLCSCs compared with qLCSCs (Figure 1(g)). Altogether, we identified and isolated SETD4-positive qLCSCs from clinical NSCLC specimens.

### 3.2. SETD4-Positive qLCSCs Were Associated with Advanced-Stage Disease in NSCLC Patients

**Figure 2:**
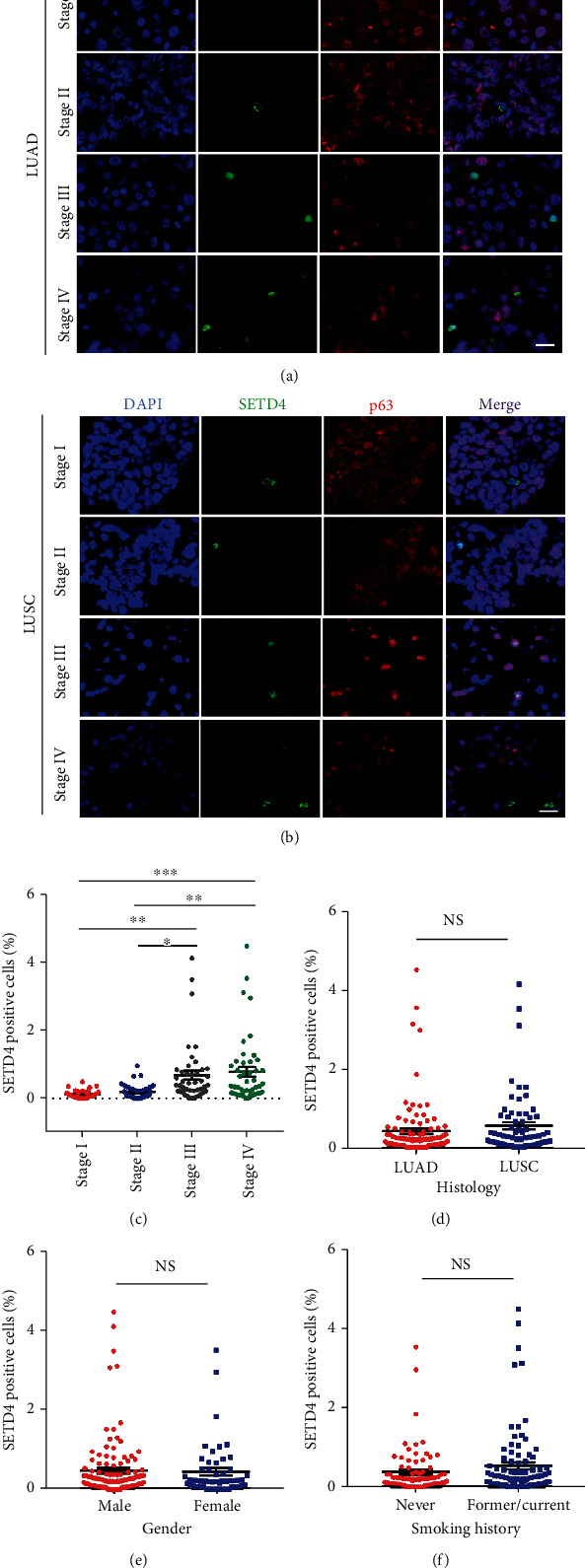
SETD4 expression in clinical lung cancer specimens is highly associated with advanced-stage disease. Representative IF images of SETD4/TTF-1 (a) from patients with different stages of LUAD and SETD4/p63 (b) from patients with different stages of LUSC. DAPI, nuclear counterstaining. Scale bar, 20 *μ*m. (c) Statistical analysis of the proportion of SETD4-positive cells in tumor tissues from NSCLC patients at different stages. Statistical comparisons were performed using a two-tailed Student's *t* test. ^∗∗∗^*p* < 0.001; ^∗∗^*p* < 0.01; ^∗^*p* < 0.05. Statistical analysis of the proportion of SETD4-positive cells in tumor tissues from patients with different histological types (d), sexes (e), and smoking histories (f). NS: no significant. The data are presented as the mean ± SEM.

We then identified the level of SETD4-positive cells in tumor samples obtained from 166 NSCLC patients. The clinical characteristics of these patients are shown in Table 1. Figures 2(a) and 2(b) show representative IF images of SETD4 in different stages of LUAD (Figure 2(a)) and LUSC (Figure 2(b)). The proportion of SETD4-positive qLCSCs in tumor tissues from advanced-stage (stages III and IV) patients (0.68 ± 0.13% and 0.78 ± 0.14%, respectively) was significantly higher than that in tumor tissues from early-stage (stages I and II) patients (0.12 ± 0.02% and 0.19 ± 0.04%, respectively) (Figure 2(c)). However, there were no significant differences in the proportions of SETD4-positive qLCSCs in patients with different histological types (Figure 2(d)), sexes (Figure 2(e)), or smoking histories (Figure 2(f)).

### 3.3. SETD4-Positive qLCSCs Are Highly Associated with Chemoresistance in NSCLC Patients

**Figure 3:**
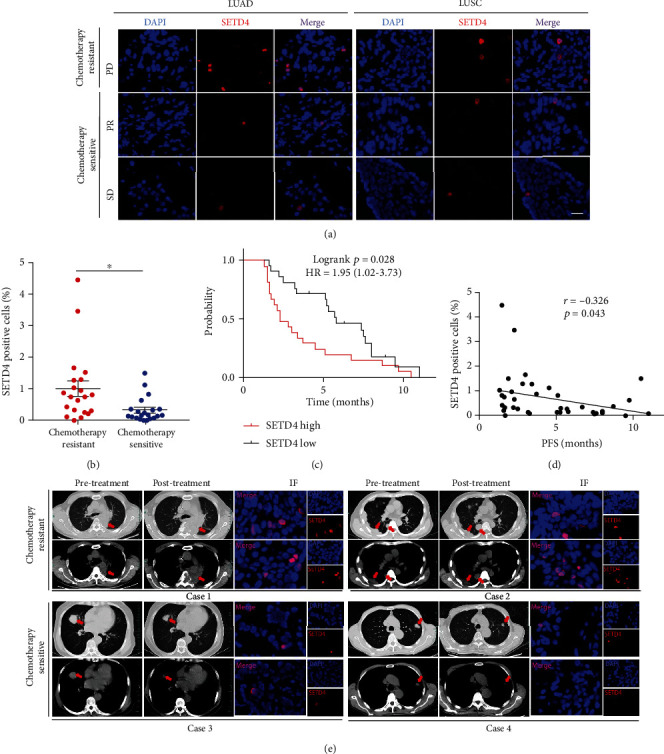
The proportion of SETD4-defined qLCSCs was correlated with chemoresistance in clinical lung cancer patients. (a) Representative IF images of SETD4 in chemotherapy-resistant patients and chemotherapy-sensitive patients. DAPI, nuclear counterstaining. Scale bar, 20 *μ*m. (b) Proportion of SETD4-positive cells in chemotherapy-resistant patients (*n* = 21) and chemotherapy-sensitive patients (*n* = 21). The data are presented as the mean ± SEM. ^∗^*p* < 0.05. (c) Comparison of progression-free survival (PFS) between SETD4 high (*n* = 21) and SETD4 low (*n* = 21) patients (*p* = 0.028). Patients were divided into SETD4-high and SETD4 low cohorts according to the median proportion of SETD4-positive cells (0.32%). The median PFS of the SETD4 high and SETD4-low cohorts was 2.3 and 5.8 months, respectively. (d) Correlation between the proportion of SETD4-positive cells and PFS. Pearson *r* analysis showed that the proportion of SETD4-positive cells was negatively correlated with PFS (*r* = −0.326, *p* = 0.043). (e) Representative chest CT images of 2 chemotherapy-resistant patients and 2 chemotherapy-sensitive patients and the corresponding IF images of SETD4.

Since SETD4 was highly expressed in chemoradioresistant qLCSCs, we speculated that SETD4 might predict the response to chemotherapy in NSCLC patients. To verify this hypothesis, we enrolled NSCLC patients who had received platinum-based combination chemotherapy. Treatment responses were evaluated according to RECIST version 1.1 after every 2 cycles of chemotherapy. Patients who experienced PD after 2 or 4 cycles of chemotherapy were considered chemotherapy resistant, whereas patients who did not experience PD after 4 cycles of chemotherapy were considered chemotherapy sensitive. Then, we selected 21 chemoresistant patients and 21 chemosensitive patients and retrospectively analyzed the proportion of SETD4-positive qLCSCs in tumor tissue obtained before chemotherapy. The clinical characteristics of these patients are shown in Table 2. The proportion of SETD4-positive qLCSCs was significantly higher in the chemoresistant group than in the chemosensitive group (Figures 3(a) and 3(b)). Moreover, patients with more SETD4-positive qLCSCs had a shorter PFS than those with fewer SETD4-positive qLCSCs (2.3 months vs. 5.8 months, HR = 1.95 (1.02–3.73), *p* = 0.028) (Figure 3(c)). Pearson r correlation analysis revealed that PFS was negatively correlated with the proportion of SETD4-positive cells (*r* = −0.326, *p* = 0.043) (Figure 3(d)).  Figure 3(e) shows representative chest CT images for 4 patients. We observed significant enlargement of tumor lesions after 2 cycles of chemotherapy in case 1 and case 2, both of which had high expression of SETD4. Conversely, we observed tumor shrinkage in case 3 and case 4, both of which had low expression of SETD4. These results demonstrated that SETD4 is associated with chemotherapy resistance in NSCLC patients.

### 3.4. SETD4 Confers Resistance to Chemoradiotherapy by Inducing Cellular Quiescence

**Figure 4:**
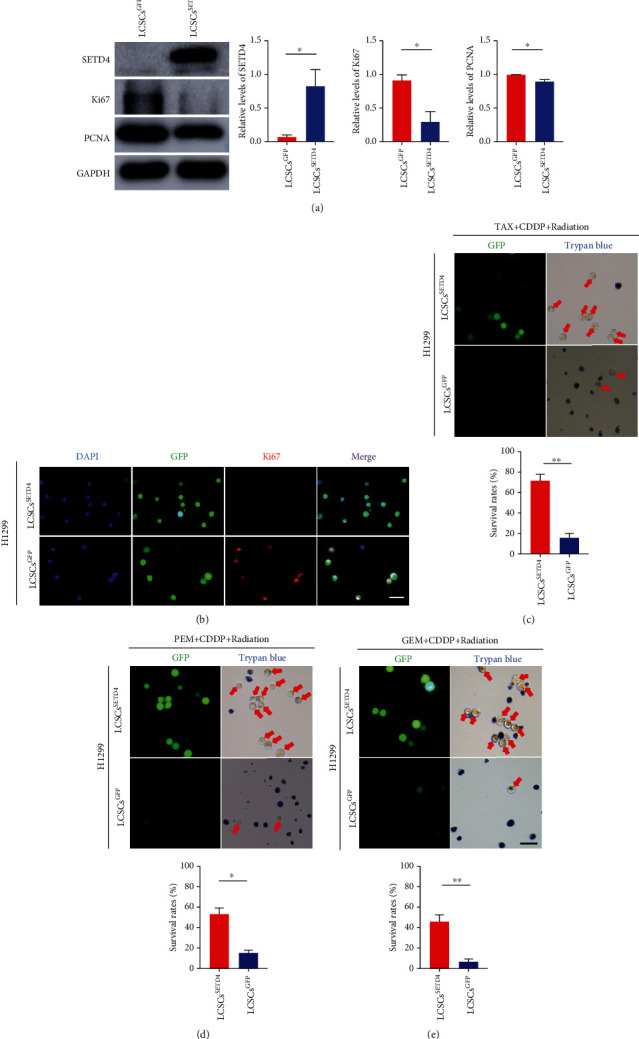
SETD4 confers resistance to chemoradiotherapy by facilitating cell quiescence. (a) Protein levels of SETD4, Ki67, and PCNA in CD133-sorted LCSCs (derived from H1299 cell lines) overexpressing GFP (LCSCs^GFP^) or SETD4 (LCSCs^SETD4^). *n* = 3. GAPDH was used as an internal reference. ^∗^*p* < 0.05. (b) Representative IF images of Ki67 in LCSCs^SETD4^ and LCSCs^GFP^. DAPI, nuclear counterstaining. Scale bar, 50 *μ*m. Representative images and survival rate analysis of LCSCs^GFP^ and LCSCs^SETD4^ after treatment with Taxol (TAX) (200 nmol/L)+CDDP (10 *μ*mol/L)+radiation (c), pemetrexed (PEM) (200 nmol/L)+CDDP (10 *μ*mol/L)+radiation (d), and gemcitabine (GEM) (0.5 *μ*mol/L)+cisplatin (CDDP) (10 *μ*mol/L)+radiation (e). *n* = 3. Scale bar, 50 *μ*m. ^∗∗^*p* < 0.01; ^∗^*p* < 0.05. The data are presented as the mean ± SD.

To further verify that chemoradiotherapy resistance is regulated by SETD4, we overexpressed SETD4 in LCSCs. LCSCs were separated from H1299 cell lines by CD133 sorting, as CD133 is a commonly used cell surface marker to isolate CSCs from lung cancer [36–38]. We observed a reduction in proliferation markers, including Ki67 and PCNA, in SETD4-overexpressing LCSCs (Figures 4(a) and 4(b)), indicating that SETD4 overexpression led to cell quiescence. In addition, a large proportion of SETD4-overexpressing LCSCs survived after 3 days of TAX (200 nmol/L) plus CDDP (10 *μ*mol/L) treatment combined with radiation (30 Gy), while the control cells (LCSCs^GFP^) were sensitive to the treatment, and extensive cell death was observed (Figure 4(c)). Similar findings were observed in LCSCs treated with radiation (30 Gy) combined with PEM (200 nmol/L) plus CDDP (10 *μ*mol/L) or GEM (0.5 *μ*mol/L) plus CDDP (10 *μ*mol/L) (Figures 4(d) and 4(e)). We also performed individual drug treatments at different concentrations and found that SETD4-overexpressing LCSCs exerted consistent drug resistance after different drug treatments including TAX (Figure S4A), PEM (Figure S4B), GEM (Figure S4C), and CDDP (Figure S4D). Additionally, a decrease in the caspase-3 signal was observed in SETD4-overexpressing LCSCs versus GFP control cells 24 hours after TAX (200 nmol/L) plus CDDP (10 *μ*mol/L) treatment (Figure S5), indicating reduced cell apoptosis after drug treatment in SETD4-overexpressing LCSCs. Thus, SETD4 confers resistance to chemoradiotherapy by inducing cell quiescence.

### 3.5. Quiescent LCSCs Suppressed Cell Proliferation and Metabolism

**Figure 5:**
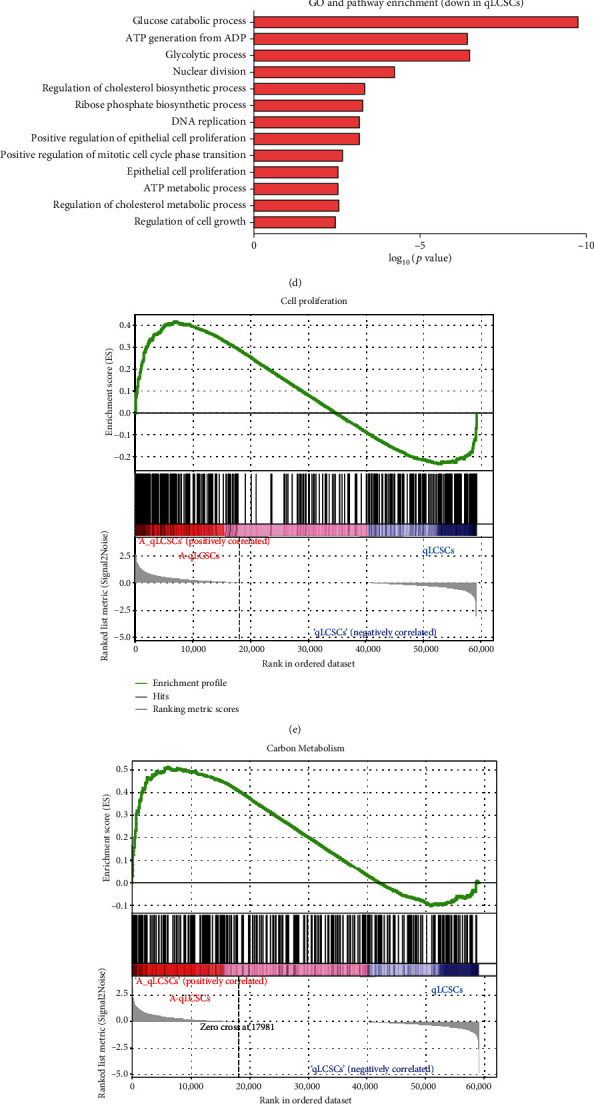
Transcriptome profile of SETD4-defined qLCSCs and A-qLCSCs. (a) Heatmap of differentially expressed genes (DEGs) between qLCSCs and A-qLCSCs.*n* = 3. (b) Volcano plot of DEGs. The red dots indicate upregulated genes, and the green dots indicate downregulated genes. Gene ontology (GO) and pathway enrichment analysis of upregulated (c) and downregulated DEGs in qLCSCs (d). GSEA showed downregulated expression of genes involved in cell proliferation (e), carbon metabolism (f), and the PI3K-AKT signaling pathway (g) in qLCSCs compared with A-qLCSCs.

To characterize the molecular signatures of SETD4-positive qLCSCs and investigate the underlying mechanism of SETD4-mediated chemoradioresistance, we performed RNA-seq analysis of qLCSCs and A-qLCSCs. Differentially expressed genes between qLCSCs and A-qLCSCs are presented in a heatmap (Figure 5(a)). A total of 1054 genes were upregulated and 774 genes were downregulated in qLCSCs (Figure 5(b)). GO enrichment analysis showed that the downregulated genes in qLCSCs were enriched in the glucose catabolic process, ATP generation from ADP, the glycolytic process, cell proliferation, and DNA replication (Figures 5(c) and 5(d)). GSEA showed downregulation of genes involved in cell proliferation (Figure 5(e)), carbon metabolism (Figure 5(f)), and the PI3K-AKT signaling pathway in qLCSCs (Figure 5(g)).

### 3.6. SETD4 Controls Cell Quiescence via H4K20me3 Catalysis in NSCLC, and PI3K-mTOR Is a Possible Downstream Target

**Figure 6:**
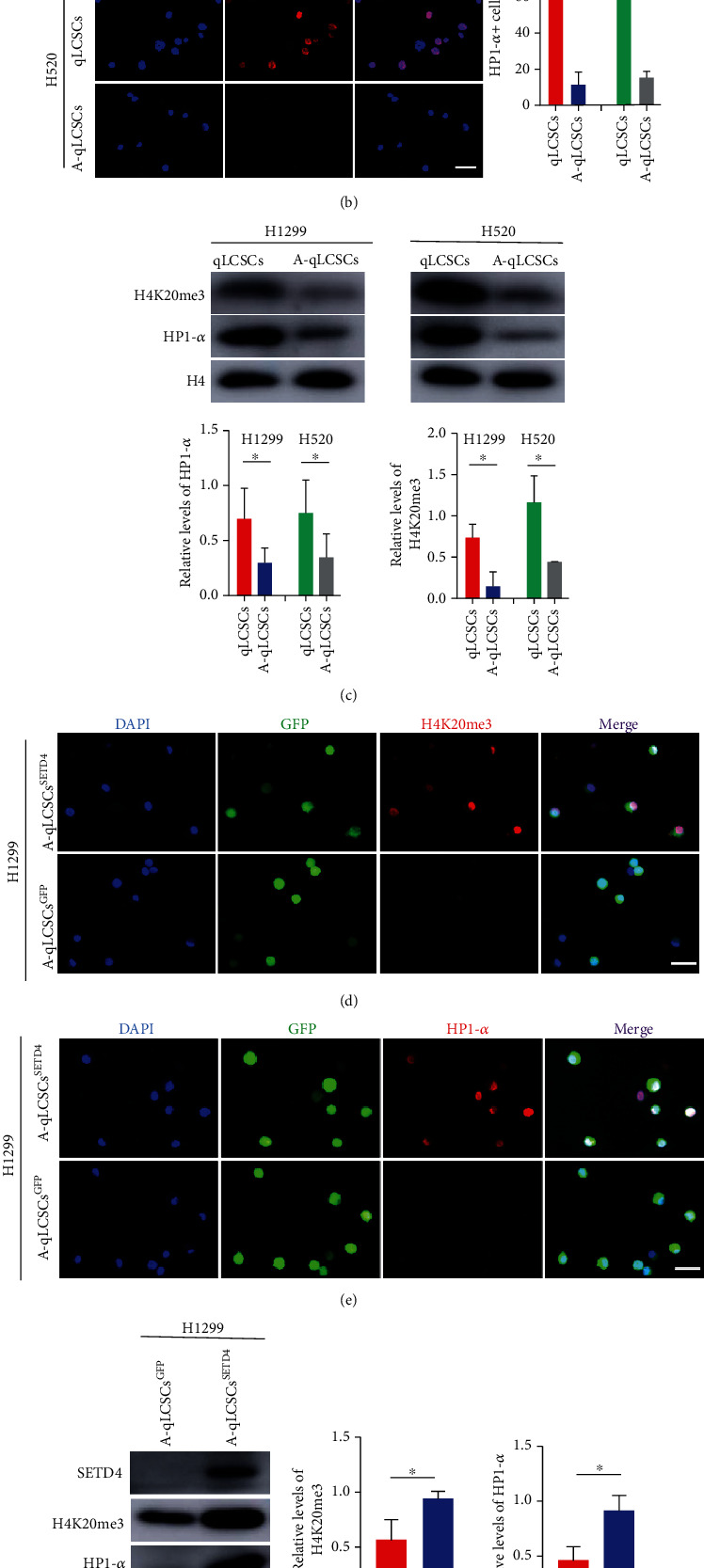
SETD4 controlled cell quiescence by inhibiting the PI3K-mTOR pathway via H4K20me3 catalysis. Representative IF images of H4K20me3 (a) and HP1-*α* (b) in qLCSCs and A-qLCSCs derived from H1299 and H520 cell lines. DAPI, nuclear counterstaining. Scale bar, 50 *μ*m. (c) Protein levels of H4K20me3 and HP1-*α* in qLCSCs and A-qLCSCs. H4 was used as an internal reference. *n* = 3. ^∗^*p* < 0.05. Representative IF images of H4K20me3 (d) and HP1-*α* (e) in A-qLCSCs^SETD4^ and A-qLCSCs^GFP^. DAPI, nuclear counterstaining. Scale bar, 50 *μ*m. (f) Protein levels of H4K20me3 and HP1-*α* in A-qLCSCs^SETD4^ and A-qLCSCs^GFP^. H4 was used as an internal reference. ^∗^*p* < 0.05. (g) Protein levels of PTEN, mTOR, PI3K, and p-PI3K in A-qLCSCs^SETD4^ and A-qLCSCs^GFP^. GAPDH was used as an internal reference. GAPDH was used as an internal reference. ^∗^*p* < 0.05. Data are presented as the mean ± SD.

We previously found that SETD4 facilitated heterochromatin formation via H4K20me3 catalysis in *Artemia* diapause embryos and BCSCs, thereby leading to cell quiescence [32, 33]. H4K20me3 was reported to play a key role in constitutive heterochromatin formation and cell quiescence [39–41], and heterochromatin protein 1-*α* (HP1-*α*) is a conserved biomarker of heterochromatin formation [42, 43]. Accordingly, we hypothesized that SETD4 controls cell quiescence in NSCLC in the same manner. Consistently, H4K20me3 and HP1-*α* were highly expressed in qLCSCs compared with A-qLCSCs (Figures 6(a)–6(c)), indicating the enrichment of constitutive heterochromatin in qLCSCs. Moreover, there was no significant change in the level of H3K9ac (Figure S6A and B), which is an indicator of chromatin. Then, we overexpressed SETD4 in A-qLCSCs, which exhibited low expression of SETD4, and we found that H4K20me3 and HP1-*α* and were markedly upregulated (Figures 6(d)–6(f)). These results demonstrated that SETD4 facilitates heterochromatin formation via H4K20me3 catalysis in NSCLC, which strengthens previous findings and reveals an evolutionarily conserved mechanism of SETD4-mediated cell quiescence in different types of CSCs.

The above results suggest that SETD4 catalyzes H4K20me3 in specific gene regions and leads to gene silencing. Based on our RNA-seq analysis, the PI3K-AKT pathway was downregulated in qLCSCs; thus, we speculated that SETD4 epigenetically regulates the PI3K-AKT signaling pathway. PTEN, a negative regulatory factor of the PI3K-AKT pathway, has been reported to regulate cell quiescence in stem cells [25]. Mammalian target of rapamycin (mTOR), downstream of PI3K-AKT, has been identified as a key factor involved in stem cell quiescence [44]. Then, we overexpressed SETD4 in A-qLCSCs and detected the protein levels of PTEN, mTOR, PI3K, and phosphorylated PI3K (p-PI3K). As shown in Figure 6(g), overexpression of SETD4 led to a significant increase in the expression of PTEN as well as a significant decrease in p-PI3K and mTOR, indicating that the PI3K-mTOR pathway is a possible downstream target of SETD4.

## 4. Discussion

In the present study, we investigated the correlation between the abundance of SETD4-positive qLCSCs and clinical stage, chemotherapy sensitivity, and prognosis in NSCLC patients. We used SETD4 to define a subgroup of chemoradioresistant qLCSCs, thus revealing ideas for treatment strategies targeting SETD4-positive qLCSCs.

Quiescent CSCs play a critical role in tumor recurrence and chemoresistance. Approximately 30% to 55% of patients with NSCLC develop recurrence or distant metastasis after surgery [45]. Even stage I NSCLC reoccurs in approximately 25% of patients after curative resection [46], and reoccurrence is currently considered to be attributable to quiescent CSCs [15]. In the present study, we confirmed the existence of a group of SETD4-positive cells, defined as qLCSCs, in tumor tissues from early-stage NSCLC patients and found that the proportion of SETD4-positive qLCSCs in advanced-stage patients was significantly higher than that in early-stage patients. Hence, SETD4 could be used as a biomarker to predict the malignancy of NSCLC. There are currently no effective biomarkers for tumor recurrence risk after surgery or postoperative adjuvant chemotherapy in patients with early-stage NSCLC. SETD4 is a biomarker of qLCSCs, yet it remains unclear whether SETD4 can predict postoperative recurrence in patients with early-stage lung cancer. Further follow-up is needed to determine the time to tumor recurrence after surgery among early-stage patients. If the expression level of SETD4 is associated with postoperative tumor recurrence, then it may serve as a potential biomarker for predicting lung cancer recurrence and metastasis.

Platinum-based chemotherapy is one of the main treatment options for advanced NSCLC patients. However, the response rates to conventional platinum-based chemotherapy range from 19% to 36% in NSCLC patients [47–49]; these rates are unsatisfactory, and resistance results in a poor prognosis. It is difficult to identify patient subpopulations who are more likely to develop chemotherapy resistance in the clinic. Previous studies have demonstrated that chemoresistance is attributed to the increased proliferation of cancer cells [50–52]. In contrast to previous studies, this study proposes that quiescent CSCs are the root population responsible for chemoresistance and that SETD4 is a unique marker of quiescent CSCs. In this study, SETD4-positive qLCSCs were examined in NSCLC patients who received platinum-based chemotherapy, and high SETD4 expression was found to be correlated with poor chemotherapy efficacy and prognosis. Thus, SETD4 may be emerging as a potential biomarker for predicting the efficacy of chemotherapy and may be a druggable target in the treatment of NSCLC. These findings provide an effective biomarker for identifying qCSCs in tumor tissues and have broad implications for clinical therapies. For patients with high levels of SETD4-positive qLCSCs, conventional chemotherapy regimens have limited therapeutic effects, and new individualized treatment strategies need to be explored further.

Studies with sample types ranging from *Artemia* embryos to human breast cancers have demonstrated the evolutionarily conserved mechanism of SETD4-regulated CSC quiescence across different species. In the present study, we found that SETD4 overexpression in CSCs derived from an NSCLC cell line led to cell quiescence and resistance to chemoradiotherapy. More importantly, we verified SETD4-mediated chemoresistance in NSCLC patients who received platinum-based chemotherapy. This is an extension of a previous study and validated that SETD4-positive qCSCs exert therapeutic resistance across different tumor types. Hence, SETD4 could be a potential therapeutic target to overcome drug resistance and might be applicable to a variety of tumors.

Quiescent CSCs can be activated under favorable conditions and divide asymmetrically into quiescent CSCs and proliferating CSCs. Proliferating CSCs divide continually and result in tumor relapse [33]. Hence, different treatment strategies should be adopted for different patients. For early-stage NSCLC patients who undergo surgery, the activation of qCSCs is a key factor that leads to tumor recurrence and metastasis after surgery. For these patients, some studies proposed that keeping these cells in a quiescent state for a long period could help prevent local progression or distant metastasis [53, 54]. Quiescence can be maintained by the suppression of proliferative signaling or the activation of quiescence-related pathways. For example, inhibitors of CDK4/6 can block the transition from the G0/G1 phase to the S phase of the cell cycle, thus inhibiting tumor growth, recurrence, and metastasis [55, 56]. On the other hand, for advanced-stage NSCLC patients who are resistant to chemotherapy, since chemotherapy treatment selects a subpopulation of chemoresistant qCSCs, strategies are urgently needed to completely eliminate these cells. Some studies have proposed that activating these cells and forcing them cells to reenter the cell cycle may sensitize them cells to chemotherapy drugs [53]. Our RNA-seq analysis revealed downregulation of genes involved in cell proliferation, glucose metabolism, and PI3K-AKT signaling pathway markers in qLCSCs than in A-qLCSCs. We thus inferred that drugs that alter the hypometabolic and low proliferative states of qLCSCs may activate these cells and that a combination of sequential chemotherapy can clear these activated cells. These hypotheses remain to be verified in future studies. Further study on the characteristics of SETD4^+^ CSCs and the epigenetic regulatory mechanism of SETD4 could provide novel insight into methods for overcoming resistance to chemotherapy and radiotherapy.

## Figures and Tables

**Table 1:** Clinicopathological features of 166 NSCLC patients.

Characteristics	Clinical stage			
	Stage I (*n* = 40)	Stage II (*n* = 36)	Stage III (*n* = 44)	Stage IV (*n* = 46)
Age (years)				
Median (range), y	66 (34-85)	68 (46-88)	66.5 (35-79)	65.5 (32-81)
Sex				
Male	24	24	35	32
Female	16	12	9	14
Smoking history				
Never	25	15	16	20
Former/current	15	21	28	26
Histology				
Adenocarcinoma	28	21	20	27
Squamous carcinoma	12	14	24	16
Adenosquamous carcinoma	0	0	0	2
Poorly differentiated NSCLC	0	1	0	1

NSCLC: nonsmall cell lung cancer.

**Table 2:** Clinicopathological features of 42 NSCLC patients who received platinum-based combination chemotherapy.

Characteristics	Response evaluation	
	Resistant (*n* = 21)	Sensitive (*n* = 21)
Age (years)		
Median (range), y	66 (52-73)	63 (35-75)
Sex		
Male	20	17
Female	1	4
Stage		
IIIB, IIIC	5	8
IV	16	13
Histology		
Adenocarcinoma	6	10
Squamous carcinoma	14	11
Adenosquamous carcinoma	1	0
Chemotherapy regimens		
TAX+platinum	8	3
PEM+platinum	6	10
GEM+platinum	6	8
DTX+platinum	1	0

NSCLC: nonsmall cell lung cancer. TAX: taxol. PEM: pemetrexed. GEM: gemcitabine. DTX: docetaxel. Platinum: cisplatin or carboplatin.

## Data Availability

The data that support the findings of this study are available upon request from the corresponding author.

## Supplementary Materials

Supplementary Materials Supplementary Figure S1: representative immunofluorescence (IF) images of SETD4/CD44 in tumor tissues from NSCLC patients. White arrow indicated SETD4-positive cells. DAPI, nuclear counterstaining. Scale bar, 20 *μ*m. Supplementary Figure S2: tumorsphere formation assay of H1299 cell lines and A-qLCSCs derived from H1299 cell lines. *n* = 3. Scale bar, 200 *μ*m. Supplementary Figure S3: representative IF images of SETD4/CD133 in qLCSCs and A-qLCSCs derived from clinical LUAD and LUSC specimens. DAPI, nuclear counterstaining. Scale bar, 50 *μ*m. Supplementary Figure S4: representative images and survival rate analysis of LCSCs^SETD4^ and LCSCs^GFP^ after treatment with different concentrations of Taxol (TAX) (100 nmol/L, 200 nmol/L, and 400 nmol/L) (A), pemetrexed (PEM) (100 nmol/L, 200 nmol/L, and 400 nmol/L) (B), gemcitabine (GEM) (0.25 *μ*mol/L, 0.5 *μ*mol/L, and 1.0 *μ*mol/L) (C), and cisplatin (CDDP) (5 *μ*mol/L, 10 *μ*mol/L, and 20 *μ*mol/L) (D). *n* = 3. Scale bar, 50 *μ*m. ^∗∗∗^*p* < 0.001; ^∗∗^*p* < 0.01; ^∗^*p* < 0.05. Data are presented as the mean ± SD. Supplementary Figure S5: representative IF images of caspase-3 in LCSCs^SETD4^ and LCSCs^GFP^ after treatments with TAX (200 nmol/L) plus CDDP (10 *μ*mol/L). DAPI, nuclear counterstaining. Scale bar, 50 *μ*m. Supplementary Figure S6: (A) representative IF images of H3K9ac in qLCSCs and A-qLCSCs derived from H1299 and H520 cell lines. DAPI, nuclear counterstaining. Scale bar, 50 *μ*m. (B) Protein levels of H3K9ac in qLCSCs and A-qLCSCs. H3 was used as an internal reference.
