# Supplementary material for: SETD4 Confers Cancer Stem Cell Chemoresistance in Nonsmall Cell Lung Cancer Patients via the Epigenetic Regulation of Cellular Quiescence
Source: Stem Cells Int. 2023 May 27;2023:7367854. doi: 10.1155/2023/7367854 (PMC10239305; doi:10.1155/2023/7367854)
Supplement: Supplementary Materials — Supplementary Figure S1: representative immunofluorescence (IF) images of SETD4/CD44 in tumor tissues from NSCLC patients. White arrow indicated SETD4-positive cells. DAPI, nuclear counterstaining. Scale bar, 20 μm. Supplementary Figure S2: tumorsphere formation assay of H1299 cell lines and A-qLCSCs derived from H1299 cell lines. n = 3. Scale bar, 200 μm. Supplementary Figure S3: representative IF images of SETD4/CD133 in qLCSCs and A-qLCSCs derived from clinical LUAD and LUSC specimens. DAPI, nuclear counterstaining. Scale bar, 50 μm. Supplementary Figure S4: representative images and survival rate analysis of LCSCsSETD4 and LCSCsGFP after treatment with different concentrations of Taxol (TAX) (100 nmol/L, 200 nmol/L, and 400 nmol/L) (A), pemetrexed (PEM) (100 nmol/L, 200 nmol/L, and 400 nmol/L) (B), gemcitabine (GEM) (0.25 μmol/L, 0.5 μmol/L, and 1.0 μmol/L) (C), and cisplatin (CDDP) (5 μmol/L, 10 μmol/L, and 20 μmol/L) (D). n = 3. Scale bar, 50 μm. ∗∗∗p < 0.001; ∗∗p < 0.01; ∗p < 0.05. Data are presented as the mean ± SD. Supplementary Figure S5: representative IF images of caspase-3 in LCSCsSETD4 and LCSCsGFP after treatments with TAX (200 nmol/L) plus CDDP (10 μmol/L). DAPI, nuclear counterstaining. Scale bar, 50 μm. Supplementary Figure S6: (A) representative IF images of H3K9ac in qLCSCs and A-qLCSCs derived from H1299 and H520 cell lines. DAPI, nuclear counterstaining. Scale bar, 50 μm. (B) Protein levels of H3K9ac in qLCSCs and A-qLCSCs. H3 was used as an internal reference. [file 7367854.f1.zip › Supplementary materials description.docx]

**Supplementary Figure S1.** Representative immunofluorescence (IF) images of SETD4/CD44 in tumor tissues from NSCLC patients. White arrow indicated SETD4-positive cells. DAPI, nuclear counterstaining. Scale bar, 20 μm.

**Supplementary Figure S2.** Tumorsphere formation assay of H1299 cell lines and A-qLCSCs derived from H1299 cell lines. n=3. Scale bar, 200 μm**.**

**Supplementary Figure S3.** Representative IF images of SETD4/CD133 in qLCSCs and A-qLCSCs derived from clinical LUAD and LUSC specimens. DAPI, nuclear counterstaining. Scale bar, 50 μm.

**Supplementary Figure S4.** Representative images and survival rate analysis of LCSCs^SETD4^ and LCSCs^GFP^ after treatment with different concentrations of Taxol (TAX) (100 nmol/L, 200 nmol/L, 400 nmol/L) **(A)**, pemetrexed (PEM) (100 nmol/L, 200 nmol/L, 400 nmol/L) **(B)**, gemcitabine (GEM) (0.25 μmol/L, 0.5 μmol/L, 1.0 μmol/L) **(C)**, and cisplatin (CDDP) (5 μmol/L, 10 μmol/L, 20 μmol/L) **(D)**. n=3. Scale bar, 50 μm. *** p<0.001, ** p<0.01, *p<0.05. Data are presented as the mean ± SD.

**Supplementary Figure S5.** Representative IF images of caspase-3 in LCSCs^SETD4^ and LCSCs^GFP^ after treatments with TAX (200 nmol/L) plus CDDP (10 μmol/L). DAPI, nuclear counterstaining. Scale bar, 50 μm.

**Supplementary Figure S6.** (**A**) Representative IF images of H3K9ac in qLCSCs and A-qLCSCs derived from H1299 and H520 cell lines. DAPI, nuclear counterstaining. Scale bar, 50 μm. (**B**) Protein levels of H3K9ac in qLCSCs and A-qLCSCs. H3 was used as an internal reference.
